# Perspectives on sperm donor anonymity: insights from donor-conceived adults in Belgium

**DOI:** 10.1093/humrep/deae160

**Published:** 2024-07-15

**Authors:** Phyline Casteels, Julie Nekkebroeck, Herman Tournaye

**Affiliations:** Brussels IVF Centre for Reproductive Medicine, UZ Brussel, Vrije Universiteit Brussel, Brussels, Belgium; Brussels IVF Centre for Reproductive Medicine, UZ Brussel, Vrije Universiteit Brussel, Brussels, Belgium; Brussels IVF Centre for Reproductive Medicine, UZ Brussel, Vrije Universiteit Brussel, Brussels, Belgium

**Keywords:** donor conception, donor anonymity, disclosure, donor, donor insemination

## Abstract

**STUDY QUESTION:**

Are donor-conceived adults in Belgium interested in obtaining donor information, and do these interests vary based on their family backgrounds?

**SUMMARY ANSWER:**

Donor-conceived adults express a significant interest in obtaining donor-related information, with the highest interest reported by offspring from heterosexual couples compared to those from lesbian couple-parented or single-parent families.

**WHAT IS KNOWN ALREADY:**

In Belgium, sperm donation is mainly anonymous, but the rise of direct-to-consumer genetic testing challenges this anonymity.

**STUDY DESIGN, SIZE, DURATION:**

This was a cross-sectional study involving an online nationwide survey conducted from July 2022 to October 2023. Participants, aged 18 years and older and being aware of their anonymous sperm donor-conceived status, were recruited through various channels.

**PARTICIPANTS/MATERIALS, SETTING, METHODS:**

A total of 203 participants were included: 62.6% grew up in heterosexual families with infertile fathers, 26.1% with lesbian couples, 8.4% with single parents, and 3.0% in various or diverse family structures. The survey was available in both French and Dutch and consisted of 43 questions, including a mix of yes/no questions and multiple-choice items.

**MAIN RESULTS AND THE ROLE OF CHANCE:**

The average age of disclosure was 16.5 years, with notably later disclosure in heterosexual couple-parented households. A substantial 82.8% of donor-conceived individuals expressed a keen interest in obtaining non-personally identifiable donor information, while 69% were curious about personally identifiable donor data. Furthermore, 61.6% conveyed a desire for personal contact with their donors, and 26.6% advocated for the inclusion of the donor’s name on their birth certificates. Participants raised in lesbian two-parent families exhibited the lowest level of interest in donor-related information compared with those raised in other family structures. An overwhelming 90.1% wondered about the possibility of having half-siblings from the same sperm donor. Analysis of survey responses on DNA database registration revealed that 55.2% of donor-conceived offspring were already registered, with 68.8% discovering the same donor offspring and 30.4% successfully locating their donors. Compared to individuals from other family structures, those raised in heterosexual couple-parented households exhibit a less positive attitude toward their conception through anonymous sperm donation. About 61.6% of donor-conceived individuals reported experiencing distinct emotions compared to their peers, while 44.1% encountered psychological difficulties related to anonymous sperm donation, primarily attributed to late disclosure. The majority supported the idea of informing the donor about the number of children he facilitated to conceive. Lastly, the study highlighted that 21.2% of donor-conceived adults considered becoming donors themselves, and 31.3% expressed willingness to use an anonymous donor whenever faced with fertility challenges.

**LIMITATIONS, REASONS FOR CAUTION:**

Our sample size may not fully represent all adults conceived through anonymous sperm donation in Belgium. Participation bias may have influenced the results, especially due to the overrepresentation of participants from heterosexual couples. Additionally, an association exists between individuals raised by heterosexual couples and late disclosure, complicating the analysis by introducing a confounding factor.

**WIDER IMPLICATIONS OF THE FINDINGS:**

The findings of this study contribute to a better understanding of the needs and preferences of donor-conceived adults, with significant potential impact on patient education and healthcare policy.

**STUDY FUNDING/COMPETING INTEREST(S):**

Study funding was not obtained for this research. There are no conflicts of interest to disclose.

**TRIAL REGISTRATION NUMBER:**

N/A.

## Introduction

It is believed that the first Belgian donor-conceived child was born in Brussels in the 1950s. However, since donor insemination was not considered an acceptable medical practice, this activity was shrouded in secrecy, and no medical records were kept. Donor sperm requests were then met by using fresh sperm. This changed in 1970 with the establishment of the first sperm banks. While artificial insemination initially addressed the desire for children among infertile heterosexual couples, it has also been extended to assistance for single women and lesbian couples since 1980 (Claes, 2022).

Historically, fertility specialists discouraged openness and only assisted heterosexual couples. Donor anonymity was implemented because of concerns about protecting family dynamics, societal stigma, legal considerations, and the historical context surrounding perceptions of biological versus social parenthood (Guttmacher *et al.*, 1950; Claes, 2022). Consequently, many donor-conceived individuals who later discover their anonymous sperm donor conception are raised in heterosexual households (Schrijvers *et al.*, 2019; Claes, 2022). Currently, early disclosure is recommended, as it is believed to facilitate the integration of this knowledge into one’s sense of identity (Rumball and Adair, 1999). Individuals who receive this information early, typically exhibit a considerably more favorable attitude toward sperm donation compared to those who receive it later in life, and experience more positive feelings toward their parents (Scheib *et al.*, 2005; Paul and Berger, 2007; Jadva *et al.*, 2009; Freeman and Golombok, 2012; Tallandini *et al.*, 2016). Late disclosure often leads to feelings of confusion, and donor-conceived individuals frequently express a desire that their parents had informed them earlier about being donor-conceived, as highlighted by Turner and Coyle (2000).

Until 2007, donor conception was a largely unregulated practice in Belgium. This means that each fertility center defined its own individual policy. Most sperm donors from that era were anonymous, and their anonymity was ensured by means of a written agreement with the clinic. As clinics were not obliged to register donor-assisted fertility procedures, accurate statistics regarding the number of donor-conceived individuals remain elusive.

A significant turning point emerged in 2007 with the enactment of the ‘Law on Medically Assisted Reproduction and the Destination of Surplus Embryos and Gametes’ (Staatsblad, 2007). It granted individuals the choice between anonymous and non-anonymous sperm donation. According to the law, non-anonymous donation involves bringing in a known donor. Anonymous donation means that neither the donor, nor the prospective parent(s), nor the resulting children can obtain information about each other. Notwithstanding the use of an anonymous donor, it is known that donor-conceived individuals have an interest in various levels of donor-related information (Turner and Coyle, 2000; Jadva *et al.*, 2009, 2010; Scheib *et al.*, 2017).

With the changing societal values and evolving family structures, especially the increasing number of single-parent and lesbian couple-parented families, openness about sperm donation within the family is becoming the norm (Beeson *et al.*, 2011). However, it appears that parents find it challenging to find appropriate language to discuss donor conception. Especially, the term used by parents and donor-conceived individuals to refer to their donor proves to be a sensitive issue (Mac Dougall *et al.*, 2007; Provoost *et al.*, 2017). Furthermore, the increasing popularity of direct-to-consumer genetic testing to uncover information about one’s ancestry has added a new dimension to the landscape of donor conception. Donor-conceived adults who have not been informed of their status may discover their genetic background through these tests and seek out their first-degree relatives, leading to potentially unexpected revelations (Harper *et al.*, 2016). Among donor-conceived individuals, there exists a certain need to discuss the donor and the experience of being donor-conceived (Widbom *et al.*, 2023). This need is evident in initiatives such as online and offline support groups or communities, where individuals in similar situations provide peer support. Research has shown that such peer support can significantly alleviate feelings of loneliness and stigma associated with being donor-conceived (Turner and Coyle, 2000; Crawshaw *et al.*, 2013).

The issue of whether to retain anonymous sperm donation is gaining increasing traction, both in the political arena and in the media. However, individuals born through this reproductive method have often been excluded from these conversations. In line with earlier research by Mahlstedt *et al.* (2010), who surveyed 85 sperm donor-conceived adults in the USA, our study aims to delve into the experiences, perspectives, and opinions of donor-conceived individuals in Belgium. Specifically, it seeks to explore the degree of interest expressed by these individuals in accessing donor information. Furthermore, we aim to investigate whether there are differing perspectives among donor-conceived individuals from different family backgrounds.

## Materials and methods

### Study design and participants

This study aimed to gather insights into the experiences, attitudes, and perspectives of adults conceived through anonymous sperm donation in Belgium. An online survey was conducted using the Qualtrics online questionnaire platform between July 2022 and October 2023. To participate, individuals had to be at least 18 years old at the time of their involvement in the study. The study-specific questions were developed based on clinical experience and feedback from relevant experts. The survey was made available in both French and Dutch and comprised 43 questions, designed to take approximately 20 min to complete. Participants were recruited through various channels, including social media, word of mouth, magazine, and newspaper articles, as well as support groups. Access to the questionnaire was provided via a web link. Obviously, only donor-conceived individuals who were aware of their method of conception took part in the study. Ethical approval for the research was obtained from the Medical Ethics Committee (UZ Brussels) on 13 April 2022. Steps were taken to ensure participant anonymity and confidentiality throughout the survey process.

### Data collection

The survey featured a combination of yes/no questions and multiple-choice items. An ‘other, please specify’ option was also available, enabling the provision of personalized responses that extended beyond the given choices, which was ideal for capturing nuanced experiences or perspectives not encapsulated within the structured options.

The questionnaire was organized into six distinct categories for a comprehensive assessment.

#### Demographic information

Survey participants provided essential background details, including their birth year and gender. They were also asked about their religious beliefs, the medium through which they came into contact with the study, their family situation, and the presence or absence of siblings within their familial unit during upbringing.

#### Disclosure and communication about donor conception

This section investigated the timing and manner in which participants were informed about their conception through anonymous sperm donation, including who disclosed the information (Mother, Father, Both parents, Other, Self-discovered) and the context of disclosure (Planned conversation, Discussion, Other). The survey inquired about the participants’ discussion partners regarding donor conception (Mother, Father, Both Parents, Peer Support Groups, Friends, Other, or Not discussed), as well as their opinions on whether donor conception can be sufficiently discussed (Yes, No) and the frequency of these discussions (Never, Rarely, Sometimes, Often, Always).

Participants were also questioned about their perceptions regarding the main reasons they were informed about their donor conception: A desire to avoid secrets, Concerns that a secret might disrupt the parent–child relationship, Others being aware, The belief that the child had a right to know the medical history, A non-taboo attitude toward donor conception, Reaching an appropriate age for disclosure, Transparency from the start due to being the child of lesbian dyad parents or a single mother, and Other. For those who found out unintentionally or were informed by individuals other than their parents, the survey inquired about the reasons participants believed their parent(s) had not disclosed the information themselves. The options included: Fear of societal rejection, Concerns about reactions to using donor conception, Perceiving the information as irrelevant, Fear that the child would reject the social father, Concerns about the child’s ability to process the information, Procrastination, Not knowing the reason, and Other. Finally, participants were prompted to reflect on their initial emotional reactions to the disclosure, choosing from a range of responses such as: Indifference, Interest, Confusion, Avoidance, Shock, Disappointment, Happiness, and Other. The long-term effects of late disclosure were also examined, with possible outcomes including: A breach of trust between parent and child, Feelings of deception, Growing up with a sense of living a lie, and Other.

#### Attitude toward knowledge about the donor and other donor relatives

Participants were queried on several aspects related to the disclosure and information about their sperm donor and donor siblings. Specifically, participants were asked if they believe that mandatory disclosure should be compulsory (Yes, No, No Opinion), and whether they wish to have access to non-personally identifiable information (non-PII) about the donor (Yes, No, No Opinion). They were also surveyed on their desire for personally identifiable information (PII) (Yes, No, No Opinion), direct personal contact (PC) with the donor (Yes, No, No Opinion), and the inclusion of the donor’s name on their birth certificate (Yes, No, No Opinion). Interest in discovering second-degree relatives was also probed (Yes, No). Further, participants were inquired whether they would register in an international DNA database (Yes, No, Already Registered), and for those who were already registered, if they had found: Second-degree relatives, The donor, or Had yet to find any matches. Questions were also posed about potential registration in a Belgian DNA database (Yes, No, No Opinion), and if affirmative, the amount they would be willing to pay for such a service. Information preferences were gauged regarding: Second-degree relatives, The donor themselves, Both or Neither. Participants’ opinions were also sought on whether they believe the donor should have rights to certain information: The number of children conceived, non-PII, PII, A conversation, Rights to information if the children are aware and agree, Rights to information if the parents are aware and agree, and The donor should not have any right to information. A corresponding set of questions was directed toward participants’ views on the rights of parents to receive information: The number of children conceived with the same donor, Non-PII, PII, No rights, Rights to information if the child receives the same information.

#### Personal consequences

In this section of the survey, participants were presented with two yes/no questions: Did you ever feel different from your peers because you were conceived using donor material? and Have you ever encountered psychological challenges as a result of being conceived through anonymous sperm donation? Those who responded affirmatively were prompted to provide further details in an open-ended response format. In the analysis of free-text responses, the chief researcher systematically reviewed all responses, identifying common themes or patterns, and subsequently grouped them into categories based on their content and relevance to the research objectives. When discussing the feeling of being different from peers, the following categories were identified and considered: Identity formation and kinship, Emotional impact and social interaction, Medical and genetic aspects, and Social acceptance and awareness. Regarding psychological difficulties, the categories delineated included: Identity crisis, Breach of trust with parents, Depression and psychological issues, Challenges in relationships, and Feelings of injustice.

#### Attitude toward donor, legal parent(s), and method of conception

Participants were asked to describe their perception of the sperm donor: Biological father, Donor, Father, Other. They were also asked to reflect on the figure they identified as their father during upbringing, with possibilities such as: Biological father, Legal father, Adoptive father, Social father, and if applicable, The absence of a father figure. The quality of the relationship with this male figure was rated on a 5-point Likert scale, from Very good to Very bad, inclusive of an option for those without a father figure. The relationship with the female figure they were raised by was similarly evaluated, and for those raised by two women, they had the opportunity to provide further detail. Finally, participants were invited to express their overall views on sperm donation, offering their opinions on a scale from Very poor to Very good.

#### Attitude toward heterologous medically assisted reproduction

Participants were queried about their preferences for the manner in which sperm donation should be conducted: Completely anonymous, Anonymous with disclosure of non-PII, Anonymous with the disclosure of PII, Non-anonymous, Prohibition of sperm donation, No opinion, and Other. For those who opted for disclosure of non-PII or PII, the survey sought to determine the age at which they believed this information should be released. Additionally, participants were asked if they would consider becoming a sperm donor themselves (Yes, No, Already a donor), and if they would contemplate the use of anonymous sperm donation in the event of unresolved desires to have children (Yes, No, Have already utilized this option, Uncertain).

### Data analysis

Participants’ birth years were organized into five decades: the 1960s, the 1970s, the 1980s, the 1990s, and the 2000s. Additionally, the age at which they were informed about their conception was classified using Erik Erikson’s stages of psychosocial development, including infancy and early childhood (0–3 years), preschool (4–6 years), school age (7–11 years), adolescence (12–18 years), young adulthood (19–40 years), and middle adulthood (41–65 years) (Erikson, 1950). Quantitative data underwent chi-square test analysis to identify any statistically significant differences and associations among the categorized variables. A significance level of *P* < 0.05 was used to determine statistical significance. A Bonferroni-adjusted significance level of *P* < 0.00109 was applied in multinomial logistic regression analyses to evaluate the nuanced interplay between family type, age at disclosure, and the participants’ interest levels in various types of donor-related information. This adjustment was implemented to minimize the elevated risk of Type I errors due to multiple comparisons. By employing this rigorous statistical approach, we were able to assess the intricate relationships between these variables while controlling for potential confounding factors. All data were processed for statistical examination using SPSS software, version 29.

## Results

### Demographic data

A total of 203 participants took part in the research: 34% male (n = 69), 65.5% female (n = 133), and one non-binary (0.5%). The majority (62.6%, n = 127) grew up in heterosexual families with an infertile father, 26.1% (n = 53) grew up with lesbian couples, 8.4% (n = 17) with a single parent, and 3% (n = 6) in various or diverse family structures (Table 1). Among all participants, 75% grew up with siblings in the same household. The largest portion of respondents, who were born in the 1980s (34.0%) and 1990s (37.4%), became aware of the study through various means, with the largest proportion (48.3%) discovering it through the Belgian Facebook group Donor Children/Donors. During their upbringing, 67.5% were raised in Christian households, while 27.6% identified as atheist or agnostic. This distribution has shifted with 22.7% currently identifying as Christians and 51.2% as atheist or agnostic.

**Table 1.** Family background and demographic characteristics.

	All (n = 203)		**HC (n = 127)**		**LC (n = 53)**		**SM (n = 17)**			**Other (n = 6)**	
	n	%	n	%	n	%	n		%	n	%
**Language**											
Dutch	193	95.1	119	93.7	53	100		16	94.1	5	83.3
French	10	4.9	8	6.3	0	0		1	5.9	1	16.7
**Gender**											
Male	69	34.0	35	27.6	26	49.1		5	29.4	3	50
Female	133	65.5	92	72.4	27	50.9		12	70.6	2	33.3
Non-binary	1	0.5	0	0	0	0		0	0	1	16.7
**Age at study**											
Mean (SD)	33.6 (8.764)		37.6 (7.885)		27.1 (5.692)			25.6 (4.690)		29.2 (6.494)	
Range	18–62		19–62		18–38			20–37		23–40	
**Age at disclosure**											
Infancy, early childhood (0–3 years)	49	24.1	6	4.7	30	56.6		13	76.5	0	0
Preschool (4–6 years)	23	11.3	4	3.1	16	30.2		2	11.8	1	16.7
School age (7–11 years)	18	8.9	11	8.7	4	7.5		1	5.9	2	33.3
Adolescence (12–18 years)	32	15.8	27	21.3	3	5.7		1	5.9	1	16.7
Young adulthood (19–40 years)	73	36.0	71	55.9	0	0		0	0	2	33.3
Middle adulthood (41–65 years)	8	3.9	8	6.3	0	0		0	0	0	0
**Siblings during upbringing**											
Yes	153	75.4	96	75.6	47	88.7		6	35.3	4	66.7
No	50	24.6	31	24.4	6	11.3		11	64.7	2	33.3
**Family change during upbringing**											
Yes	91	44.8	62	48.8	24	45.3		2	11.8	3	50
No	112	55.2	65	51.2	29	54.7		15	88.2	3	50

HC, heterosexual couple; LC, lesbian couple; SM, single mother.

### Age of disclosure about anonymous sperm donation associated with family structure

The overall age of disclosure was 16.5 years (SD 13.657). Among the respondents, 55.7% discovered later in life (defined as age 12 years or older) that they were conceived through anonymous sperm donation, with an average age of disclosure at 26.32 years (SD 10.104), ranging from a minimum age of 8 years to a maximum of 59 years. The majority of these respondents (47.4%) were born in the 1980s, 20.2% in the 1970s, and 28.9% in the 1990s. Within this group 85.8% (n = 109) were raised in heterosexual couple-parented families, 1.9% (n = 1) in lesbian two-parent families, and 5.9% (n = 1) by single parents. The average age of disclosure in heterosexual two-parent families was 23.7 years (SD 11.816). The age of disclosure has decreased across the decade of the respondent’s birth: 54.3 years (SD 8.08) in the 1960s group; 26 years (SD 13.1) in the 1970s group; 25 years (SD 10.56) in the 1980s group; 18.35 years (SD 7.47) in the 1990s group; 10 years (SD 14.14) in the 2000s group. In lesbian couple-parented households, the average age of disclosure was 3.72 years (SD 3.784), while in single-parent families, it was 2.9 years (SD 3.370) (Table 1). When this information was revealed later in life, it was primarily conveyed by the mother in 37.7% of cases, while 16.7% of individuals discovered it on their own, and 19.3% learned of it through third parties. In the single-mother and lesbian two-parent families, no-one reported discovering it on their own or through others. Among heterosexual couples, 40.2% learned about it through a planned conversation, while 59.8% found out through a discussion or unplanned means. The main reason why the offspring was informed depends on the family structure in which one has been raised: the importance of knowing the medical history (heterosexual couples) and not considering it a taboo or not wanting to keep secrets from the child (lesbian couples and single mothers). The most common reasons for parents to delay informing their children about donor conception include fears of potential negative consequences, such as community reactions (14.8%), child rejection of their (social) father (9.4%), concerns about the child having a hard time with the information (9.4%), as well as uncertainty about the timing of disclosure (7.4%). Among respondents who learned about their conception later in life, the majority reported feeling confused (29.1%) and shocked (28.6%), with long-term experiences of growing up with a sense of deception (39.4%). There was no statistically significant difference in these reactions between cases where this information was provided through a planned conversation or through unplanned means. Individuals from lesbian couples and single-parent households who learned about their conception later in life (n = 2) expressed different reactions, such as indifference or curiosity, rather than confusion or shock.

### Communication about being donor conceived

Participants were asked, ‘Do you think there can be sufficient discussion about donor conception?’ with 41.9% (n = 85) expressing the belief that this topic was sufficiently discussed. Notable disparities were observed across various family structures: 27.6% among heterosexual couples, 77.4% among lesbian couples, 47.1% among single mothers, and 16.7% among individuals from other family structures (χ^2^ = 40.874, *P* < 0.001). For those who believe there can be enough discussion, 8.1% (n = 7) never discuss this topic, while 36% (n = 31) discuss it a few times per year, 45.3% (n = 39) discuss it about monthly and 10.5% (n = 9) discuss it about weekly. These conversations are primarily held with friends (69.5%, n = 141) and support groups (37.9%, n = 77). A significant majority of participants overall, i.e. 84.7% (n = 172), believe that children conceived through anonymous sperm donation should be informed about this. However, 8.9% (n = 18) of participants held a contrary view, with 7.9% of participants who grew up with heterosexual couples opposing the idea of disclosure and 13.2% of participants who grew up with lesbian couples expressing the same viewpoint. Notably, all participants (n = 18) raised by single mothers expressed a desire for mandatory disclosure. Additionally, 6.4% of participants did not express a definitive opinion on the matter (χ^2^ = 15.214, *P* = 0.012).

### Desires toward knowing (about) the donor and considerations

In the survey, participants were given a list of answers to describe their donor and were asked to mark all the terms that matched their view. The majority uses the word ‘donor’ (68%, n = 138). This is particularly the case for individuals raised by lesbian couples (90.6% n = 48) and single mothers (94.1% n = 16). Only 55.1% (n = 70) of those raised by heterosexual couples used the word donor, while 55.9% (n = 71) of them used the term ‘biological/genetic father’. These differences among various family structures are statistically significant (for donor χ^2^ = 31.477, *P* < 0.001; for biological father χ^2^ = 20.418, *P* < 0.001). Age also influenced terminology: older individuals or those who learned of their donor conception at an older age were more inclined to use terms like ‘biological/genetic father’. Among individuals who referred to the male contributor as ‘donor’, 40% express strong negative feelings about anonymous sperm donation. In contrast, among those who used the term ‘biological/genetic father’, 78.1% reported such negative feelings. Regarding the male guardian, he was primarily perceived as a ‘legal father’ (38.9% n = 79), followed by a ‘social father’ (28.6% n = 58). Five of the participants were raised by lesbian couples and two were raised by single parents had a father figure in their upbringing.

In donor-conceived individuals raised in various family structures, a significant 82.8% (n = 168) expressed a desire to know non-PII about the donor, with the highest percentage (92.1%, n = 117) among those raised by heterosexual couples (χ^2^ = 29.315, *P* < 0.001) (Table 2). Individuals from lesbian couples and single-mother households also expressed substantial interest at 60.4% (n = 32) and 76.5% (n = 13), respectively. The overall curiosity for PII was 69.0% (n = 140), with the highest interest among those raised by heterosexual couples (82.7%, n = 105), 52.9% (n = 9) among those raised by single mothers, and 39.6% (n = 21) among those raised by lesbian couples (χ^2^ = 39.359, *P* < 0.001). Among all participants, 61.6% (n = 125) expressed a desire for PC with their donors, with higher rates among individuals raised in heterosexual two-parent families (78.0%, n = 99) and single mothers (47.1%, n = 8). In lesbian couple-parented families, this was only 26.4% (n = 14) (χ^2^ = 51.920, *P* < 0.001). Additionally, 26.6% (n = 54) of donor-conceived individuals believe that the donor’s name should be on their birth certificate, particularly with a significant percentage among those raised in heterosexual couple-parented families (34.6%, n = 44) (χ^2^ = 39.869, *P* < 0.001). Moreover, 90.1% (n = 183) wonder about the possibility of having genetic relatives of the second degree, with substantial interest across family structures, including heterosexual couples (95.3%, n = 121), lesbian couples (77.4%, n = 41), and single mothers (94.1%, n = 16) (χ^2^ = 12.606, *P* = 0.003) (Table 2).

**Table 2.** Overview of respondents’ preferences and beliefs regarding obtaining information about the sperm donor and genetic background, categorized by family type.

Have you ever wished to know non-personally identifiable information (non-PII) about the donor?										
Family type	HC (n = 127)		LC (n = 53)		SM (n = 17)		Other (n = 6)		All (n = 203)	
	n	%	n	%	n	%	n	%	n	%
No	8	6.3	20	37.7	4	23.5	0	0	32	15.8
Yes	117	92.1	32	60.4	13	76.5	6	100	168	82.8
No opinion	2	1.6	1	1.9	0	0	0	0	3	1.5
**Have you ever wished to know personally identifiable information (PII) about the donor?**										
No	18	14.2	31	58.5	8	47.1	1	16.7	58	28.6
Yes	105	82.7	21	39.6	9	52.9	5	83.3	140	69.0
No opinion	4	3.1	1	1.9	0	0	0	0	5	2.5
**If possible, would you wish for personal contact (PC) with the donor?**										
No	18	14.2	35	66.0	8	47.1	1	16.7	62	30.5
Yes	99	78.0	14	26.4	8	47.1	4	66.7	125	61.6
No opinion	10	7.9	4	7.5	1	5.9	1	16.7	16	7.9
**Do you believe that the name of you donor should be on your birth certificate?**										
No	57	44.9	46	86.8	14	82.4	3	50.0	120	59.1
Yes	44	34.6	4	7.5	3	17.6	3	50.0	54	26.6
No opinion	26	20.5	3	5.7	0	0	0	0	29	14.3
**Have you ever wondered if you have genetic relatives in the second degree (half-siblings)?**										
No	6	4.7	12	22.6	1	5.9	1	16.7	20	9.9
Yes	121	95.3	41	77.4	16	94.1	5	83.3	183	90.1

HC, heterosexual couple; LC, lesbian couple; SM, single mother.

In multinomial regression, those who rate their maternal relationship as ‘good’ (χ^2^ = 527.366, *P* < 0.001) or ‘very good’ (χ^2^ = 3872.251, *P* < 0.001) strongly prefer non-PII. While, males (χ^2^ = 652.876, *P* < 0.001) and those with strong maternal bonds (χ^2^ = 181.002, *P* < 0.001) are less interested in PII. Amongst males raised by lesbian couple-parented families, we observe less interest in PII (58.1%) compared to those raised by heterosexual couple-parented families (38.9%) and single mothers (12.5%). Individuals born in the 1960s, 1970s, and 1980s showed more of a tendency for PC, however, this trend lacks statistical significance. A sub-analysis among donor-conceived individuals raised by heterosexual couples revealed no significant variables related to (non)-PII and PC with the donor.

Regarding interest in second-degree relatives, 90.1% (n = 183) of the participating donor-conceived individuals expressed a desire to obtain information. No statistically significant variables were identified.

### DNA database registration

Of the donor-conceived individuals, 55.2% (n = 112) were already registered in an international DNA database, and an additional 21.7% (n = 44) expressed willingness to register. There was a statistically significant difference (χ^2^ = 58.935, *P* < 0.001) observed among various family structures, with 54.7% (n = 29) of individuals raised in lesbian two-parent households showing reluctance to register. Among those who were registered, 68.8% (n = 77) have discovered second-degree relatives, and 30.4% (n = 34) have located their donor, with 27.7% (n = 31) of them also identifying both, while 28.6% (n = 32) had not yet found any matches.

The majority, comprising 77.3% (n = 157) of the participants, were open to registering their DNA in a Belgian DNA database. Among them, 13.4% (n = 21) expressed interest in connecting with same-donor relatives, 12.7% (n = 20) in identifying their donor, and 70.1% (n = 110) in both. Donor-conceived individuals are willing to invest an average of €119.90 (SD 419.760) for such a test, with a median cost of €50.

### Psychological aspects

Compared to individuals raised in heterosexual couple-parented families, where only 24.2% (n = 30) expressed positive feelings about their method of conception, we observed significantly higher rates among those from lesbian dyad households and single-parent families, with 77.4% (n = 41) and 52.9% (n = 9) respectively reporting positive sentiments. When children were informed about their conception at a young age (infancy and preschool), they tended to exhibit a markedly more positive attitude toward sperm donation compared to those who are informed at a later stage in life (χ^2^ = 79.872, <0.001). Additionally, participants were asked whether they ever felt different from their peers based on being conceived using donor material. Our data reveal that 61.6% (n = 125) of donor-conceived individuals experienced such feelings. These feelings significantly differ based on family structure: 71.7% (n = 91) among those raised by heterosexual couples, 34% (n = 18) among those with lesbian couple families, and 70.6% (n = 12) among those with single mothers (χ^2^ = 22.844, *P* < 0.001). Participants were provided with a free text box to further elucidate their experiences, revealing that these feelings primarily impacted aspects of identity formation and kinship, such as feeling distinct due to the absence of a father figure or experiencing a sense of incompleteness. Moreover, participants were asked a yes/no question about whether they had ever experienced psychological difficulties related to being conceived through anonymous sperm donation; 44.1% (n = 89) responded affirmatively, with 24.3% specifying these difficulties as identity crises. Notably 78.7% (n = 70) of those who experienced psychological challenges were informed later in life about their donor conception. The variability in these reports was statistically significant across different family structures: 57.1% (n = 72) among heterosexual couples, 11.3% (n = 6) among lesbian couples, and 41.2% (n = 7) among single mothers (χ^2^ = 36.971, *P* < 0.001). Males reported experiencing fewer psychological difficulties than females (χ^2^ = 17.893, *P* < 0.001) and there was a statistically significant relationship between delayed disclosure and increased psychological difficulties (χ^2^ = 31.668, 0.028). Participants who reported a lack of open discussion about donor conception were more likely to report experiencing psychological difficulties related to donor conception (χ^2^ = 8.115, *P* = 0.04), while those who indicated feeling similar to their peers were less likely to report psychological difficulties (χ^2^ = 9.983, *P* = 0.002).

### Legal and ethical considerations

The majority (57.6%, n = 117) of donor-conceived adults agree that the donor should be informed about how many children were born out of his donation(s), which was significantly different from the number who do not hold this view (χ^2^ = 14.239, *P* = 0.003). It was primarily the individuals raised in lesbian couple-parented families who did not share this view (64.2% n = 34). Furthermore, 35% (n = 71) of respondents believe that the donor should have access to non-PII of the children he conceived (χ^2^ = 10.137, *P* = 0.019), while 19.2% (n = 39) believe the donor should have access to PII (χ^2^ = 17.603, *P* = 0.002). In the event of this information being shared with the donor, 68% (n = 138) of donor-conceived adults expressed a desire to be informed and advocate for their consent before any disclosure (χ^2^ = 11.492, *P* = 0.008). Similar patterns emerged when considering the possibility of parents acquiring donor information. Approximately 54.7% (n = 111) of donor-conceived adults preferred parents to be informed about the number of children conceived from one donor (χ^2^ = 11.708, *P* = 0.010). Additionally, 49.8% (n = 101) believe that parents have the right to access non-PII (χ^2^ = 3.381, *P* = 0.340), and 28.1% (n = 57) believe that parents have the right to access PII (χ^2^ = 12.107, *P* = 0.007). The timing of this disclosure for the donor and the parents was not addressed in the study.

If donor-conceived adults were given the authority to reshape the legal framework of sperm donation, 42.9% (n = 85) would opt for donors who are not anonymous from conception. Among these respondents, 49.6% (n = 61) were from heterosexual couples. For those raised in lesbian two-parent families, 38.5% (n = 20) favored anonymity with the option to release non-PII at a specific age, while 36.5% (n = 19) preferred anonymity with the option to release PII at a designated point in time. The opinions of children raised by single mothers closely aligned with those raised by lesbian couples, with 58.8% (n = 10) and 47.1% (n = 8) respectively, choosing the aforementioned options.

In terms of the suggested ages for the release of information, the average age for non-PII to be disclosed was 12.25 years (SD 6.467), with a median of 14 years. For PII, the average suggested age was 14.31 years (SD 5.687), with a median of 16 years.

A notable 21.2% (n = 42) of donor-conceived adults have contemplated the possibility of becoming donors themselves. This inclination is most pronounced among offspring raised by lesbian couples (40.4% n = 21), in contrast to 13% (n = 16) from heterosexual couple and 17.6% (n = 3) from single-mother households. These findings demonstrate statistical significance (χ^2^ = 17.431, *P* = 0.006). Two participants, both raised in heterosexual households, had already donated gametes themselves. It is not clear whether they donated before or after they found out they were conceived through donor semen.

When asked if they would consider the use of an anonymous donor in case they faced challenges in fulfilling their desire for children, 31.3% (n = 62) expressed willingness to use an anonymous donor. This choice varied among different family backgrounds, with 17.9% (n = 22) in heterosexual couple-parented upbringing, 61.5% (n = 32) in lesbian couple-parented upbringing, and 35.3% (n = 6) in single-mother households being willing. These differences were statistically significant (χ^2^ = 58.233, *P* < 0.001). One participant was raised in a heterosexual household and two from a lesbian couple-parented upbringing had already utilized donor gametes. Additionally, 25.8% (n = 51) of participants were uncertain about whether they would pursue this option.

## Discussion

The findings in this study shed light on the perspectives of adults conceived through anonymous sperm donation in Belgium. We explored multiple dimensions, including the role of age of disclosure, attitudes toward knowledge about donors and relatives, psychological difficulties of being conceived by anonymous sperm donation, attitudes toward donors, legal aspects, and methods of conception, as well as attitudes toward heterologous medically assisted reproduction.

### Heterosexual-couple-parented families

In our study, most donor-conceived adults were raised in heterosexual-couple-parented families. Born in a period before advanced reproductive technologies like ICSI became commonplace, and where fertility specialists recommended discretion to protect the social father’s role, many only discovered their origins in later life (Schrijvers *et al.*, 2019; Indekeu *et al.*, 2021; Claes, 2022). Our data show that 86% of those who learned of their donor conception later in life grew up in heterosexual dyad families, despite this family type representing 63% of the overall participants. The existence of a social father figure often precluded questions about parentage, contrasting with the early inquiries present in single-mother and lesbian-couple families due to the absence of a father figure (Jadva *et al.*, 2009). When donor-conceived adults were asked why they believed their parents did not inform them earlier, their responses often revolved around concerns related to potential negative consequences. These concerns included fears of negative community reactions, the possibility of the child rejecting their social father, as well as concerns about the child’s readiness to comprehend the information, and uncertainty about the appropriate timing for disclosure. These findings are consistent with previous research (Cook *et al.*, 1995; Nachtigall *et al.*, 1998; Lindblad *et al.*, 2000).

Furthermore, one-third of adults from heterosexual couples discovered the details of their conception independently or through others. This highlights the importance of informing parents that even if they choose not to disclose, such information may still be revealed through alternative means (Harper *et al.*, 2016; Crawshaw, 2018). The realization of being donor-conceived led to a sense of distinctiveness from peers for over half of the individuals, impacting their identity formation and familial relationships. Early disclosure is suggested to aid in integrating this aspect into their identity (Rumball and Adair, 1999). Our study corroborates findings that delayed information can result in psychological difficulties (Ilioi *et al.*, 2017). Upon learning about their conception, the majority reported feeling confused and shocked, with no significant difference between those who had a planned conversation about their conception and those who discovered it unintentionally. Furthermore, as previously demonstrated (Turner and Coyle, 2000; Jadva *et al.*, 2009; Hertz *et al.*, 2013), with early disclosure, individuals typically exhibit a considerably more favorable attitude toward sperm donation compared to those who receive this information later in life.

Although there is a recognized need among donor-conceived individuals to discuss the donor and the experience of being donor-conceived, as mentioned by Widbom *et al.* (2023), only one in three from heterosexual couples felt they have enough opportunities for such discussions. This likely stems from the fact that most individuals in heterosexual families discover their donor-conceived status in their twenties or later, which might limit their ability to have open conversations, especially if other family members are not yet aware of their origins.

Adults conceived through sperm donation within heterosexual couple-parented families tend to favor the term ‘biological/genetic father’ rather than ‘donor’ when referring to their sperm donor. The frequency of use of this term becomes more prominent among those who are older and is more frequently used by those who learned about their donor conception at an older age. These findings are consistent with these of Hertz *et al.* (2013) and may underscore a more traditional perspective on fatherhood (Barth, 2023). Individuals who opt for the term ‘donor’ often exhibit more favorable attitudes toward anonymous sperm donation compared to those who use the term ‘genetic/biological father’.

Offspring from heterosexual couples not only exhibited the highest interest in donor-related information with more than one-third wishing for the donor’s name to be included on their birth certificate. Previous research showed that desire of donor-conceived individuals to learn about the donor is driven by various factors, including curiosity about physical and behavioral similarities, expanding their identity, the need to acquire information about hereditary diseases, and a wish to have a sense of control, which might indicate a need to fully embrace their status as donor-conceived and gain a deeper understanding of themselves (Scheib *et al.*, 2017; Indekeu *et al.*, 2021; Macmillan *et al.*, 2021; Lampic *et al.*, 2022; Widbom *et al.*, 2023). Considering the contrasting findings of Slutsky *et al.* (2016) and Lozano *et al.* (2019) regarding the influence of parent–child relationships on curiosity about donor conception, our study found that supportive maternal relationships lead to more curiosity about non-PII but less interest in PII. Individuals born in the 1960s, 1970s, and 1980s displayed a pronounced preference for personal contact with their sperm donors. This trend may be linked to an increased valuation of genealogical knowledge and essential health information, which becomes more significant as individuals progress through various life stages, a phenomenon observed by Hertz *et al.* (2013).

### Lesbian couple-parented families

In line with the research of Scheib *et al.* (2003) and Jadva *et al.* (2009), our study similarly found that lesbian couples and single parents tend to discuss donor conception more openly than heterosexual two-parent couples. This may also explain why offspring from lesbian couples find that they have sufficient opportunity to openly discuss their donor-conceived status, often bringing up the topic in conversations with friends several times a year to monthly. These donor-conceived adults report that their parents informed them because they did not consider it taboo and valued knowing the medical history.

It is more common for them to refer to the sperm donor as ‘donor’, a term that acknowledges the biological link without assigning a paternal role. This preference, as observed in the research of Hertz *et al.* (2013), is consistent with the non-traditional family structures that these individuals grow up in, where there is a deliberate shift away from the traditional labels of ‘fatherhood’. These individuals, not having a father figure, feel little compulsion to ascribe relational attributes to the donor. The ‘donor’ remains a donor, representing a kind act rather than a familial bond (Hertz *et al.*, 2013). Remarkably, one in five also made use of the term ‘genetic/biological father’.

While the majority of adults born to lesbian couples through donor conception are curious about non-PII, there is a notable drop in the desire for more personal details and direct contact with the donor. This reduced curiosity might be rooted in the strong, equitable bonds formed within their family unit, beyond biological ties (Raes *et al.*, 2015). Additionally, Vanfraussen *et al.* (2003) highlighted the possibility that the feelings toward the non-biological parent could influence this lesser interest in donor information. Men from lesbian-parented families exhibited less interest in PII than those from heterosexual-dyad and single-parent families, a trend that contrasts with the research of Scheib *et al.* (2017), which reported an even gender distribution in requests for open-identity sperm donor information among same-sex female families.

Around one in five donor-conceived adults had contemplated becoming donors, a consideration that was more common among those with lesbian dyad parents. When faced with reproductive challenges, one in three donor-conceived adults were open to using an anonymous donor themselves, while a quarter were still deciding. This pattern is consistent with current research (Siegel *et al.*, 2022) and is most notably observed in individuals raised by lesbian couples.

### Single-parent families

Individuals raised in single-mother families often receive information about their conception at a young age. This may be attributed to the unique maternal role in the child’s life, coupled with the perception that honesty about their conception is more straightforward in single-parent households devoid of paternal figures (Jadva *et al.*, 2009). Furthermore, it is pertinent to consider that single mothers may be particularly attuned to social stigmas surrounding donor conception (Zadeh *et al.*, 2013). Concurrently, individuals from single-mother households all wished for mandatory disclosure of the child’s conception. This stance contrasts with the views of adults raised in lesbian couple-parented or heterosexual couple-parented households, where nearly one in 10 participants did not deem this disclosure obligatory.

Contrary to previous research (Scheib *et al.*, 2003, 2005; Jadva *et al.*, 2009), our study revealed that offspring from single-mother families tend to use the term ‘donor’ more frequently to describe their sperm donor than those from heterosexual couple-parented families.

There was a considerable interest in both non-PII and PII in single-mother-raised individuals. Around half of them also expressed a desire for personal contact with their sperm donor. This might be due to a heteronormative bias as their mothers often would have preferred to have children in a traditional family structure, potentially shaping their children’s understanding of what family means (Bock, 2000; Zadeh *et al.*, 2013). Additionally, with fewer family members to identify with, such as a missing co-parent, they may pursue knowledge of their donor to better understand themselves (Scheib *et al.*, 2005).

### Navigating the legal framework

Regardless of their family composition, donor-conceived individuals exhibit a strong interest in connecting with their same donor offspring, a trend also noted by Scheib *et al.* (2020). Half of them had registered with an international DNA database to obtain information about their donor and same donor offspring. This reflects the global trend toward individual genetic testing and the fading of assured anonymity (Harper *et al.*, 2016). While our findings indicate a significant success rate in locating genetic relatives, it is important to consider the potential for overlapping results as half-siblings participating in this study might also have registered in the same DNA database. While our research did not delve into whether individuals learned about their donor conception via DNA databases, a survey in Germany among adults conceived through anonymous sperm donation and raised in heterosexual dyad households found that one out of 10 uncovered their origins via direct-to-consumer DNA testing (Bauer and Meier-Credner, 2023). This underscores the critical need for resources and informed discussions within the donor-conceived community and for stakeholders about the implications of using online DNA databases (Gilman *et al.*, 2024).

If donor-conceived adults could modify the current legal Belgian framework of sperm donation, more than half would opt for non-anonymous donors. Interestingly, among these respondents, a significant percentage were from heterosexual couples. Among children raised in lesbian couple-parented households, anonymity with the option to release (non)-PII at a specific age, was preferred. The opinions of children raised by single mothers closely align with those of lesbian couples. In terms of the suggested ages for the release of information, the average age suggested for disclosing non-PII is around 12 years, while for PII, it is around 14 years. This is an important finding toward any future legal initiative in our country.

Most donor-conceived adults believe that the donor should be informed about how many children he has helped to conceive. However, a significant percentage of children raised in lesbian dyad households did not share this view. Their understanding of family and kinship is less reliant on biological connections and more on the chosen, intentional structure of their family unit. Consequently, they may not feel as strong a need for biological ties or for the donor to be informed about his offspring (Hertz *et al.*, 2013). When it comes to providing information to the donor, the majority wanted to be informed about this and believe that the offspring should give their consent before any information is disclosed. Similar patterns emerge when considering parents acquiring donor information. Most donor-conceived adults prefer parents to be informed about the number of children conceived from one donor, and half believe that parents have the right to access non-PII.

### Limitations of the study

It is important to acknowledge several limitations of this survey. Despite the relatively large research group (n = 203), the study’s sample may not fully represent the entire population of adults conceived through anonymous sperm donation in Belgium. Participants with strong feelings or significant experiences related to their conception may have been more inclined to participate, potentially introducing a bias into the results. Additionally, there is a significant skew in the proportion of participants raised in different family structures, with the majority born into heterosexual couples (n = 127), while 53 were raised by lesbian couples, and 17 by single mothers, possibly limiting the ability to draw meaningful conclusions about these specific groups. Additionally, an association exists between individuals raised by heterosexual couples and late disclosure, complicating the analysis by introducing a confounding factor. Despite efforts to mitigate this through multinomial regression analysis, it is important to acknowledge that bias may persist. Nonetheless, the study offers insights into a period when society primarily endorsed heterosexual relationships and discouraged openness in fertility practices. Nowadays, the landscape is evolving with an increasing prevalence of alternative family structures, such as those headed by single parents and lesbian couple-parented families, reflecting a shift in societal norms and practices (Golombok, 2017; Claes, 2022).

The study was conducted in both Dutch and French, and language and cultural factors may have influenced responses. The majority of participants were Dutch-speaking, with only a small number of French-speaking participants (10 in total). Additionally, the study focused on individuals who were conceived in Belgium and thus subject to Belgian legislation regarding sperm donation. Nevertheless, participants may have grown up in different countries. Cultural norms and attitudes regarding donor conception can vary within Belgium and across different countries. Therefore, our findings may not be directly applicable to regions with diverse cultural norms and practices concerning sperm donation.

The data collection period from July 2022 to October 2023 may not fully capture the evolution of attitudes and experiences over time. Moreover, the reliance on self-reported information introduces the potential for recall bias, as participants may not accurately recall or report events from their past, such as the age at which they learned about their conception or their emotional status at that time. Furthermore, the survey’s design, including question-wording and response options, may have influenced participant responses. The inclusion of ambiguous or leading questions could have introduced a measurement error. Participants might have been inclined to provide responses they considered socially desirable, particularly when addressing sensitive topics like donor conception and disclosure.

This study offers a snapshot of attitudes and experiences at a specific point in time. To gain a more comprehensive understanding of how attitudes and experiences evolve, future research should consider longitudinal data tracking changes over time. Nevertheless, we envision this study as a foundational step for further exploration in this field, especially considering the changing societal attitudes and norms surrounding donor conception.

## Conclusion

This study, consistent with prior research by Mahlstedt *et al.* (2010), examines donor-conceived individuals in Belgium. Early revelation of donor conception is generally regarded as advantageous, whereas delayed disclosure can result in psychological challenges. Offspring from heterosexual couples show a heightened emphasis on the donor’s role, indicating a greater need for donor information compared to those from lesbian couple-parented or single-parent families. Furthermore, a significant portion of donor-conceived individuals express a strong desire to obtain various levels of donor-related information, a possibility currently limited by the existing Belgian legislation. To circumvent this limitation, half of the respondents had already registered with international DNA databases, with many having successfully identified a genetic relative through this method. Consequently, donor anonymity has essentially become obsolete. Given the opportunity, many donor-conceived adults would opt for non-anonymous donors and endorse the release of donor information at specific stages, including allowing donors to be aware of the number of children they have helped conceiving. Additionally, a notable percentage of donor-conceived individuals have contemplated becoming donors themselves, with the highest inclination observed among those raised by lesbian two-parent families.

## Data Availability

The data underlying this article will be shared on reasonable request to the corresponding author.
